# Seroprevalence for the tick-borne relapsing fever spirochete *Borrelia turicatae* among small and medium sized mammals of Texas

**DOI:** 10.1371/journal.pntd.0006877

**Published:** 2018-10-29

**Authors:** Brittany A. Armstrong, Alexander Kneubehl, Aparna Krishnavajhala, Hannah K. Wilder, William Boyle, Edward Wozniak, Carson Phillips, Kristen Hollywood, Kristy O. Murray, Taylor G. Donaldson, Pete D. Teel, Ken Waldrup, Job E. Lopez

**Affiliations:** 1 Department of Pediatrics, National School of Tropical Medicine, Baylor College of Medicine, Houston, Texas, United States of America; 2 Department of Molecular Virology and Microbiology, Baylor College of Medicine, Houston, Texas, United States of America; 3 Department of Biological Sciences, Mississippi State University, Starkville, Mississippi, United States of America; 4 Texas State Guard, Medical Brigade, Uvalde, Texas, United States of America; 5 Texas Department of State Health Services, Zoonosis, El Paso, Texas, United States of America; 6 Texas Department of State Health Services, Zoonosis, Midland, Texas, United States of America; 7 Department of Entomology, Texas A&M AgriLife Research, Texas A&M University, College Station, Texas, United States of America; University of Connecticut Health Center, UNITED STATES

## Abstract

**Background:**

In low elevation arid regions throughout the southern United States, *Borrelia turicatae* is the principal agent of tick-borne relapsing fever. However, endemic foci and the vertebrate hosts involved in the ecology of *B*. *turicatae* remain undefined. Experimental infection studies suggest that small and medium sized mammals likely maintain *B*. *turicatae* in nature, while the tick vector is a long-lived reservoir.

**Methodology/principal findings:**

Serum samples from wild caught rodents, raccoons, and wild and domestic canids from 23 counties in Texas were screened for prior exposure to *B*. *turicatae*. Serological assays were performed using *B*. *turicatae* protein lysates and recombinant *Borrelia* immunogenic protein A (rBipA), a diagnostic protein that is unique to RF spirochetes and may be a species-specific antigen.

**Conclusions/significance:**

Serological responses to *B*. *turicatae* were detected from 24 coyotes, one gray fox, two raccoons, and one rodent from six counties in Texas. These studies indicate that wild canids and raccoons were exposed to *B*. *turicatae* and are likely involved in the pathogen’s ecology. Additionally, more work should focus on evaluating rodent exposure to *B*. *turicatae* and the role of these small mammals in the pathogen’s maintenance in nature.

## Author summary

In arid regions of the southern United States and Mexico, tick-borne relapsing fever is primarily caused by *Borrelia turicatae*. The tick vector, *Ornithodoros turicata*, feeds indiscriminately on a variety of vertebrates; however, it is unclear which animals are competent hosts for *B*. *turicatae*. This study evaluates the exposure of small and medium sized mammals in Texas to *B*. *turicatae* and identifies likely hosts for the pathogens. This work will provide insight regarding mammals to target for surveillance to identify endemic foci and to better prevent human exposure.

## Introduction

Tick-borne relapsing fever (RF) is primarily caused by spirochetes in the genus *Borrelia* and the pathogens are transmitted when infected *Ornithodoros* ticks feed on a competent vertebrate host. In the United States and Mexico, there is an association between *Ornithodoros* ticks and RF spirochete species where *Ornithodoros hermsi*, *Ornithodoros parkeri*, *Ornithodoros turicata*, and *Ornithodoros talaje* transmit *Borrelia hermsii*, *Borrelia parkeri*, *Borrelia turicatae*, and *Borrelia mazzottii*, respectively [1]. Furthermore, these tick species involved in human disease are distributed in varying ecological niches. For example, the ecology of *O*. *hermsi* is associated with coniferous forests at elevations above 900 meters throughout the western United States and Canada [2–6]. *Ornithodoros parkeri* has also been collected in semi-arid regions of the western United States at elevations from sea level to over 2,000 meters [7, 8]. *Ornithodoros turicata* is found in arid regions of Mexico, the mid- and southwestern United States from California to Texas, and a population exists in Florida [9–11]. The ecology of *O*. *talaje* overlaps that of *O*. *turicata* and collections have occurred in Mexico and in Texas [1, 12, 13]. Of the *Ornithodoros* species that transmit RF spirochetes, *O*. *turicata* and *O*. *talaje* are currently the only ones known in Texas, yet there are few records of *O*. *talaje* collections and *B*. *mazzottii* has not been isolated in the laboratory.

The biology of both RF spirochetes and their tick vector have posed challenges in defining the pathogens’ ecology. *Ornithodoros* species are rapid feeding ticks that reside in cavities including wood crevices, dens, nests, and karst formations [6, 7, 9, 14]. Thus, the vector is rarely found attached on the vertebrate host. Moreover, in the vertebrate host, spirochetes replicate in the blood reaching densities of ~1 x 10^7^ bacteria per ml before being cleared by an antibody mediated response [15]. The pathogens undergo antigenic variation and subsequently repopulate the blood [15]. This dynamic between antigenic variation and the host antibody response can continue for two to three months in a competent host [11, 16]. The cyclic nature of RF spirochetes within a competent host poses challenges when attempting to directly detect the pathogens in the blood of wild caught animals because there are quiescent periods when the spirochetes are undetectable. Since RF spirochetes induce a robust IgG response [17–19], serological surveillance is a practical approach toward defining the pathogens’ ecology given the temporal persistence of generated antibodies in the host’s blood.

The ecology of *B*. *turicatae* is poorly defined and in this current study we utilized a diagnostic antigen, the *Borrelia* immunogenic protein A (BipA), which has been used to assess canine, rodent, and human exposure to the pathogen [17, 20]. Aside from the closely related *B*. *parkeri*, BipA is highly variable between species of RF spirochetes [20]. Moreover, a BipA homologue has not been identified in other viral, parasitic, or bacterial pathogens [17]. Utilizing this diagnostic antigen, we evaluated the exposure of wild and domestic canids, raccoons, and rodents to *B*. *turicatae*. Serum samples were collected from 23 counties in Texas and screened against *B*. *turicatae* protein lysates and recombinant BipA (rBipA). Most rodents were also identified to species by morphology and molecular sequencing of the *cytochrome B* gene. Our findings indicate that *Canis latrans* (coyote), *Urocyon cinereoargenteus* (gray fox), *Procyon lotor* (raccoon), and *Peromyscus leucopus* (white-footed mouse) may be vertebrate hosts for *B*. *turicatae* in nature.

## Methods

### Ethics statement

Rodent collections were approved by the Institutional Animal Use and Care Committee at Mississippi State University (IACUC protocol #11–091) and Texas Parks and Wildlife (Scientific Research Permit #SPR-0812-958). Collections of coyotes, gray fox, and raccoon serum samples originally occurred as part of the rabies surveillance program by the Texas Department of State Health Services. Collection of shelter canine serum samples were approved by the University of Texas Health Science Center Animal Welfare Committee (AWC-07-147 and AWC-03-029).

### Animal trapping

Animal samplings occurred between 2005 and 2018. Rodents were captured alive using Sherman live traps (H.B. Sherman Traps, Tallahassee, FL). Traps were placed in and around houses, barns, and fields in the late afternoon and baited with dried oats. The following morning traps were collected, and the animals processed. Animals were euthanized by inhalation of isoflurane and exsanguinated by cardiac puncture. A drop of blood was placed on a microscope slide and the presence of spirochetes was evaluated by dark field microscopy. Peripheral blood smears were also made on microscope slides. The remaining blood was centrifuged at 1,000 x g and serum separated from the blood clot. Animals were evaluated for argasid ticks.

Shelter dogs and wild canids and raccoons were also sampled. Serum samples from stray domestic dogs located in Brownsville, TX were collected, as previously described [21]. Coyote, gray fox, and raccoon serum samples were collected as part of the Texas Department of State Health Services rabies surveillance program. The animals were captured in Tomahawk traps, terminally sampled, and serum samples stored at -20°C.

### Mammalian identification

Canids and raccoons were identified to the species level using morphological characteristics. Rodents were identified by morphological characteristics and molecular analysis of the *cytB* gene. For rodent morphological characteristics, body weights were recorded with Pesola spring scales (PESOLA SG, Baar, Switzerland), gender determined, and body and tail measurements recorded. Photographs of each animal were obtained for future reference. For molecular analysis, a 3-mm tissue biopsy was collected from each animal, stored in 90% ethanol, and DNA extracted using the DNeasy Blood and Tissue kit (Qiagen Sciences, Inc., Germantown, MD). PCR was performed using forward (5’-CCATGAGGACAAATATCCTTCTGAGGG-3’) and reverse (5’-GCCCTCAGAAGGATATTGTCCTCATGG-3’) primers for *cytB*, and sequencing performed as previously described [19, 22]. Sequences were assembled into overlapping contiguous DNA segments (contigs) using Vector NTI 11.0 software (ThermoFisher Scientific, Waltham, MA). Contigs were evaluated using BLASTn on NCBI.

### Serological assays

Immunoblotting was performed to evaluate seroconversion against *B*. *turicatae* protein lysates and rBipA, as previously described [17]. Briefly, protein lysates from 1 x 10^7^ *B*. *turicatae* spirochetes and 1 µg of rBipA were loaded into the wells of Mini-PROTEAN TGX precast gels (Bio-Rad, Hercules, CA). Gels were run for 1.5 hours and proteins were transferred onto Immobilon PVDF membranes (Millipore, Billerica, MA). Membranes were blocked overnight with Tropix Iblock (Thermo Fisher Scientific, Waltham, MA) and then probed for one hour at room temperature with serum samples diluted 1:200. The secondary molecule was HRP-conjugated protein G (Thermo Fisher Scientific, Waltham, MA) for canids and rodents at a 1:4,000 dilution. Raccoon serum samples were probed with a goat anti-raccoon IgG-HRP conjugated antibody (Alpha Diagnostics Intl. Inc., San Antonio, TX) at a 1:4,000 dilution. The substrate used to detect binding was Amersham ECL Western Blotting Detection Reagent (GE Healthcare, Buckinghamshire, UK). A sample was considered positive for *B*. *turicatae* if we detected reactivity to at least five proteins in the *B*. *turicatae* protein lysate and rBipA.

### Ecoregion mapping

**Fig 1:**
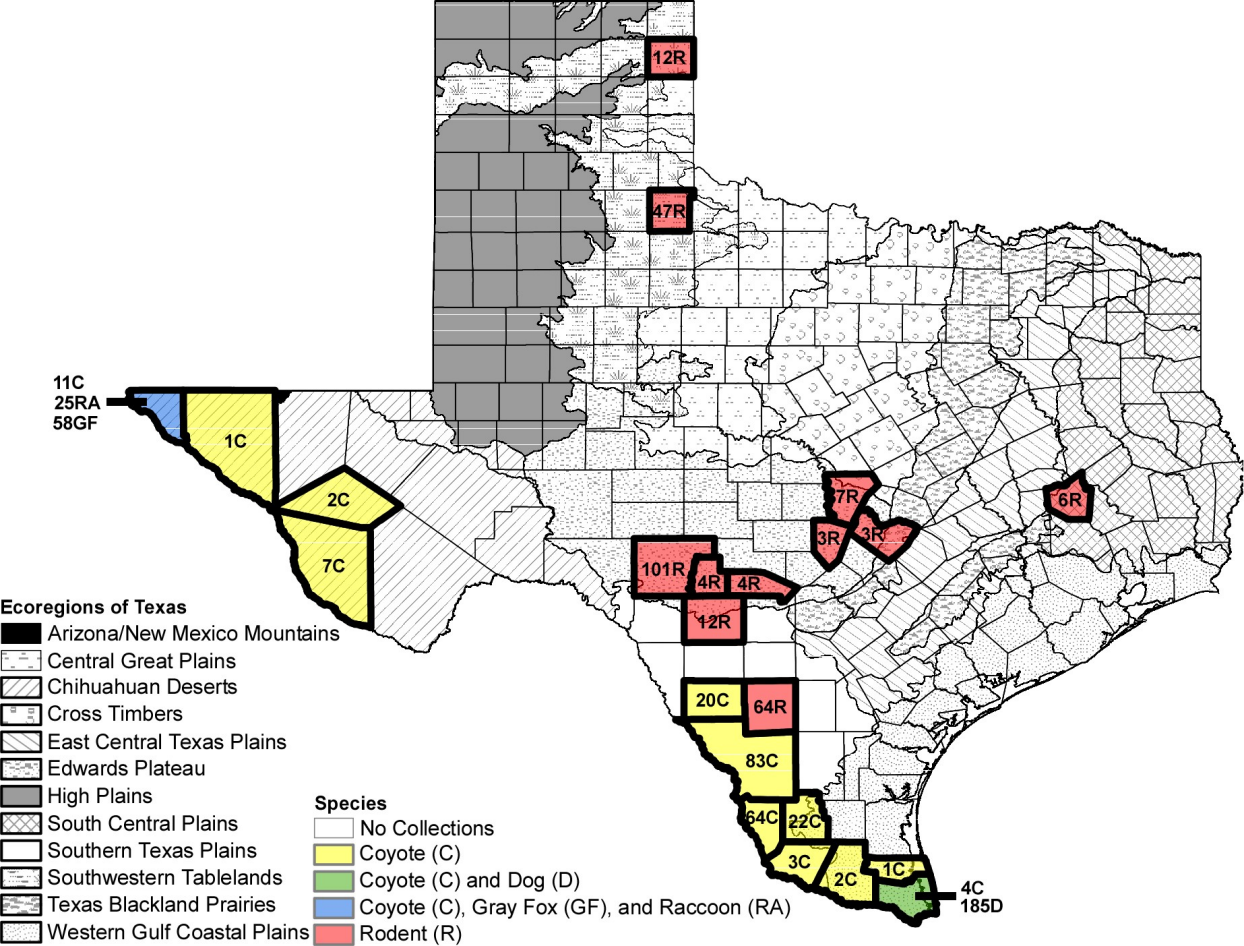
Ecoregions of Texas and counties of collection sites. Map of the 12 ecoregions of Texas overlaid with the 23 counties where collections occurred of coyote (C), dog (D), gray fox (GF), raccoon (RA), and rodents (R).

For visualizing the ecoregions of Texas we obtained a shapefile from the United States Environmental Protection Agency, which included the following 12 ecoregions: Arizona/New Mexico Mountain, Central Great Plains, Chihuahua Deserts, Cross Timbers, East Central Texas Plains, Edwards Plateau, High Plains, South Central Plains, Southern Texas Plains, Southwestern Tablelands, Texas Blackland Prairies, and Western Gulf Coastal Plains [23]. These ecoregions were defined based upon several biotic and abiotic factors such as climate, vegetation, soil type, geology, land use, wildlife, and hydrology [23]. This shapefile was then imported into ArcMap and we overlaid each county where collections occurred in Texas noting the taxa group (coyote = C, Dog = D, gray fox = GF, raccoons = RA, and rodents = R) and the number of collections (Fig 1).

### Statistics

Statistical analysis was performed using R 3.3.1 (R foundation for Statistical Computing, Vienna, Austria, https://www.R-project.org/). The 95% confidence intervals (CI) were determined for each group of samples tested that had at least one positive sample, using the proportions test. A binomial distribution was assumed with determining CI.

## Results

One to four field sites were sampled in 23 counties of Texas between 2005 and 2018. Sites included private property that was accessible through the Texas Ecolab Program and Texas Parks and Wildlife Management Areas. Counties where samples were collected were within the following Texas ecoregions: Central Great Plains, Chihuahuan desert, Cross Timber, East Central Texas Plains, Edwards Plateau, South Central Plains, Southern Texas Plains, Southwestern Tablelands, Texas Blackland Prairies, and Western Gulf Coastal Plains (Fig 1). A total of 463 canids were sampled and included 185 shelter dogs (*Canis lupus familiaris*), 220 coyotes (*C*. *latrans*) and 58 gray foxes (*U*. *cinereoargenteus*) (Fig 1). Serum samples were also collected from 25 raccoons (*P*. *lotor*) and 263 rodents (Fig 1). Argasid ticks were not detected on the animals.

**Fig 2:**
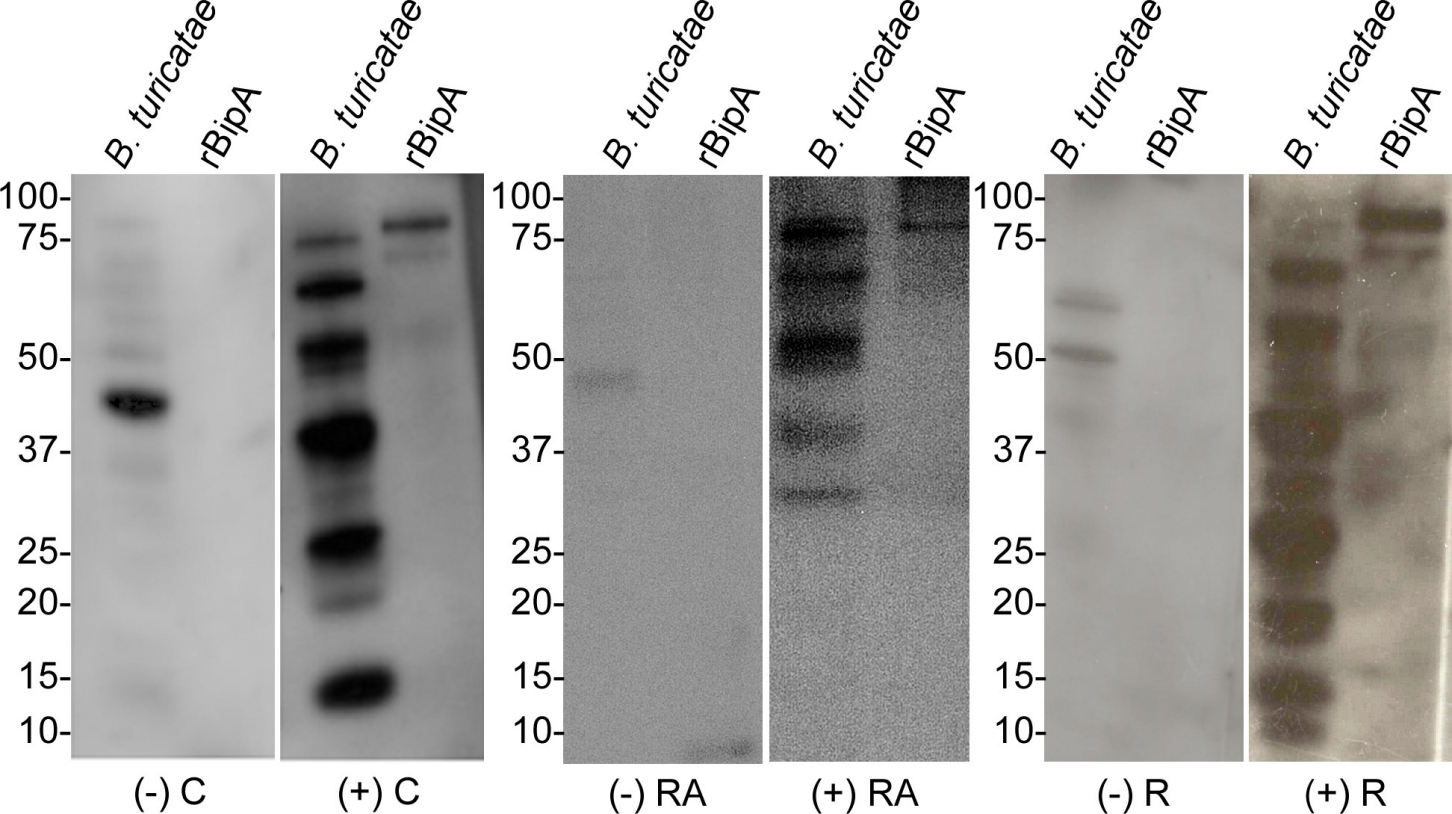
Serological evaluation of coyotes, raccoons and rodents to *B*. *turicatae* protein lysates and rBipA. Serum samples from a coyote that was considered negative (-C) and one that was positive for *B*. *turicatae* protein lysates and rBipA (+C). Also shown are immunoblots from raccoons that were considered negative (-RA) and serologically positive for *B*. *turicatae* and rBipA (+RA). Lastly, immunoblots for a negative (-R) and positive rodent (+R). Molecular masses are shown to the left of each immunoblot.

Animals were considered susceptible to infection by *B*. *turicatae* based on serological reactivity to at least five bands in *B*. *turicatae* protein lysates and rBipA. Assessing serological responses of canids (Fig 2) indicated a total seroprevalence of 5.4% (CI = 3.6–8.0%) (Table 1). None of the 185 shelter dogs that were screened had a detectable antibody response in the diagnostic assay, while 10.9% and 1.7% (CI = 7.3–16.0% and 0.09–10.5%) seroprevalence was detected in coyotes and gray fox, respectively. Webb County had the highest number of seropositive coyotes with a total of 10 animals. Presidio and Zapata County each had five seropositive animals, while Dimmit County had four. In the animals exposed to *B*. *turicatae*, a gender difference was not detected. In El Paso County, there was a single juvenile male gray fox that was seropositive, resulting in 1.7% (CI = 0.7–53.3%) prevalence among gray fox.

**Table 1:** Collection counties and years, age, sex, seroprevalence, and CI of canid samples.

County (n)	Collection Year	Age	Sex	Seroprevalence	CI
**Shelter dogs**					
Cameron (185)	2007–2009	Juvenile	Unknown	0	
**Coyote**					
Cameron (4)	2005	Adult (4)	Male (1)	0	
	2006		Female (3)	0	
Dimmit (20)	2005	Adult (20)	Male (12)	25.0% (3)	6.7–57.2
			Female (8)	12.5% (1)	0.7–53.3
El Paso (11)	2017	Adult (10)	Male (6)	0	
			Female (4)	0	
		Juvenile (1)	Male (1)	0	
			Female (0)	0	
Hidalgo (2)	2005	Adult (2)	Male (1)	0	
			Female (1)	0	
Hudspeth (1)	2009	Adult (1)	Male (1)	0	
			Female (0)	0	
Jeff Davis (2)	2018	Unknown (2)	Male (2)	0	
			Female (0)	0	
Jim Hogg (22)	2005–2006	Adult (20)	Male (8)	0	
			Female (12)	0	
	2006	Unknown (2)	Male (2)	0	
			Female (0)	0	
Presidio (7)	2008	Adult (1)	Male (0)	0	
			Female (1)	0	
	2018	Unknown (6)	Male (2)	50.0% (1)	9.5–90.5
			Female (4)	100% (4)	39.6–100
Starr (3)	2006	Adult (3)	Male (1)	0	
	2005		Female (2)	0	
Webb (83)	2005–2006	Adult (82)	Male (49)	12.2% (6)	5.1–25.5
			Female (33)	12.1% (4)	4.0–29.1
	2006	Juvenile (1)	Male (1)	0	
			Female (0)	0	
Willacy (1)	2006	Adult (1)	Male (1)	0	
			Female (0)	0	
Zapata (64)	2005–2006	Adult (63)	Male (34)	5.9% (2)	1.0–21.1
			Female (29)	10.3% (3)	2.7–28.5
		Unknown (1)	Male (0)	0	
			Female (1)	0	
**Gray Fox**					
El Paso (58)	2009, 2014–2015	Adult (30)	Male (11)	0	
			Female (19)	0	
	2014–2015	Juvenile (16)	Male (8)	12.5% (1)	0.7–53.3
	2009, 2014–2015		Female (8)	0	
	2009, 2014	Unknown (12)	Male (5)	0	
			Female (7)	0	
Total (463)				5.4% (25)	3.6–8.0

Between 2014 and 2016, raccoons were sampled in El Paso County (Table 2). One female and one male, both adults, were considered positive for *B*. *turicatae* protein lysate and rBipA (Fig 2). Both raccoons were sampled in a residential area, and a total seroprevalence of 8% (CI = 1.4–27.5%) was detected.

**Table 2:** County and collection year, age, sex, seroprevalence, and CI of sampled raccoons.

County (n)	Collection Year	Age	Sex	Seroprevalence	CI
El Paso (25)	2014–2016	Adult (12)	Male (6)	0	
			Female (6)	16.7% (1)	0.9–63.5
	2015	Juvenile (9)	Male (6)	0	
			Female (3)	0	
	2014	Unknown (4)	Male (2)	50% (1)	9.5–90.5
			Female (2)	0	
Total (25)				8% (2)	1.4–27.5

Species within seven genera of rodents were collected between 2012 and 2015 including *Peromyscus maniculatus*, *Peromyscus leucopus*, *Chaetodipus hispidus*, *Sigmodon hispidus*, *Neotoma albigula*, *Perognathus*, and *Dipodomys* species. Evaluating serological responses (Fig 2) indicated that 0.4% (CI = 0.02–2.4%) were seropositive (Table 3). *Peromyscus leucopus* was the only positive animal and originated from Edwards County.

**Table 3:** County, species, seroprevalence, and CI of sampled rodents.

County (n)	Collection Year	Species	Seroprevalence	CI
Bandera (4)	2012	*P*. *maniculatus* (3)	0	
		*S*. *hispidus* (1)		
Blanco (3)	2012	*P*. *maniculatus* (3)	0	
Burnett (7)	2012	*P*. *maniculatus* (6)	0	
		*S*. *hispidus* (1)	0	
Cottle (47)	2013	*P*. *leucopus* (2)	0	
		*P*. *maniculatus* (32)	0	
		*S*. *hispidus* (2)	0	
		*N*. *albigula* (2)	0	
		*D*. *elator* (2)	0	
		*Perognathus* spp (7) *	0	
Edwards (101)	2012	*P*. *leucopus* (22)	4.5% (1)	0.2–24.9
		*P*. *maniculatus* (58)	0	
		*S*. *hispidus* (4)	0	
		*N*. *albigula* (16)	0	
		*Dipodomys* spp (1) *	0	
Hemphill (12)	2013	*P*. *maniculatus* (7)	0	
		*P*. *leucopus* (1)	0	
		*N*. *albigula* (1)	0	
		*Dipodomys* spp (2)	0	
		*C*. *hispidus* (1)	0	
La Salle (64)	2012	*P*. *leucopus* (36)	0	
		*P*. *maniculatus* (4)	0	
		*S*. *hispidus* (10)	0	
		*N*. *albigula* (8)	0	
		*Perognathus* spp (6) *	0	
Real (4)	2012	*P*. *maniculatus* (4)	0	
Travis (3)	2012	*P*. *maniculatus* (3)	0	
Uvalde (12)	2015	*N*. *albigula* (6)	0	
		*P*. *maniculatus* (4)	0	
		*S*. *hispidus* (2)	0	
Walker (6)	2012	*P*. *maniculatus* (6)	0	
Total (263)			0.4% (1)	0.02–2.4

* Rodents were classified to the genus level.

examining blood specimens by dark field microscopy failed to detect spirochetes.

## Discussion

In this study, we began to define the ecology of *B*. *turicatae* in Texas by assessing serological responses as an indicator of host competency. While RF spirochete infections can persist for several months in a competent host [11], the pathogens’ life cycle is recurrent and direct detection of infection can be challenging because of the brevity of time when spirochetes are detectable in the blood. To circumvent this, we indirectly detected exposure to *B*. *turicatae* by assessing the vertebrate antibody response. Our findings indicate that wild canids are likely a host for *B*. *turicatae* in west Texas. These studies were also the first known serological evaluation of rodents and raccoons to *B*. *turicatae* and provided verification that exposure is occurring in this tick-host-pathogen relationship.

Rodents and insectivores are known reservoir hosts for at least two species of RF spirochete [3, 19, 24, 25], but the role of these small mammals in the ecology of *B*. *turicatae* is vague. In high elevation regions of the Western United States, sciurid rodents are the primary vertebrate host for *B*. *hermsii*, while the pathogens have also been detected in *Neotoma macrotis* [3, 24]. In regions of western Africa, *Borrelia crocidurae* is maintained in *Mastomys* and *Crocidura* species [19]. Previous tick transmission studies of *B*. *turicatae* to laboratory mice suggest that wild rodents may be susceptible to infection [17, 26, 27], and the identified seropositive *P*. *leucopus* from this current study indicated that white-footed mice are a potential competent host. However, we sampled the field site where this animal was collected three more times from 2012 to 2014 and failed to identify other positive rodents. Additional studies are needed to investigate the life cycle of *B*. *turicatae* in rodents to determine whether the pathogen attains densities in the animals that will facilitate spirochete acquisition and colonization of the tick vector.

There is mounting evidence that canids likely support the maintenance and dissemination of *B*. *turicatae* in nature. For example, the competency of domestic canines for *B*. *turicatae* was demonstrated as nearly half of the *B*. *turicatae* isolates have originated from sick dogs [28]. Moreover, successful infection of *B*. *turicatae* to a laboratory dog by tick bite suggested that the spirochetes attained sufficient densities in the blood to infect ticks [17]. In this current report, *B*. *turicatae* positive coyote and gray fox serum samples originated from Dimmit, Presidio, Webb, Zapata, and El Paso County, all of which border Mexico. With the broad home range of coyotes, these mammals are likely circulating *B*. *turicatae* between the United States and Mexico.

Coyotes possess highly organized social systems even in urban settings and are classified as transient or resident based on their territorial range [29, 30]. Transient coyotes are typically solitary subordinate young adults with a home range of 40 km^2^ to 395 km^2^. Resident coyotes have a home range of 8 km^2^ to 29 km^2^ and are part of the larger pack that include breeders, juveniles, and pups [30]. Coyote dens are often found in or around urban settings and with the expansion of these areas in Mexico and the United States, coyotes and humans are commonly in contact [30, 31].

A knowledge gap in the ecology of *B*. *turicatae* is a poor understanding regarding the dissemination of the vector in nature. *Ornithodoros turicata* are rapid feeders, completing a bloodmeal within five to 60 minutes after attachment [26]. However, it is unclear whether some proportion of ticks remain on the wild vertebrate host after engorgement, either attached or unattached, allowing for increased dissemination. Interestingly, we have collected engorged *Carios kelleyi* nymphs, which are rapid feeding argasid ticks of bats [9]. This suggests that some argasid species may remain on the vertebrate host for a duration of time after feeding. Population genetic studies are needed to evaluate the genetic diversity between *O*. *turicata* populations at different spatial scales collected in the United States, to estimate dissemination patterns of both vector and pathogen.

A limitation of our study is the likely circulation of additional uncharacterized RF spirochete species in the southern United States. While BipA is highly divergent between most species of RF spirochete and the recombinant protein can discriminate between *B*. *hermsii* and *B*. *turicatae* infections [17], additional work is needed to obtain novel spirochete species circulating in nature. For example, *Ornithidoros talaje* was recently collected in Texas [1], and while we failed to detect *Borrelia* DNA in these the ticks, the circulation of *Borrelia mazzottii* in the state exists. In 1955, *B*. *mazzottii* was reported to be transmissible by *O*. *talaje* ticks that were collected in northern Mexico [12], but since then reports of the disease have been absent. Recently, RF spirochetes were detected in a blood smear of a sick patient in Sonora, Mexico, but the species was unidentified [32]. Furthermore, Candidatus *Borrelia texasensis* was initially isolated in medium from an adult ixodid tick, *Dermacentor variabilis*, which was feeding on a coyote collected in Webb County, Texas [33]. The spirochete was initially cultured and grouped with RF spirochetes, but Lin and colleagues were unable to revive frozen stocks and an isolate does not exist. While it is unclear whether coyotes are a competent host for Candidatus *Borrelia texasensis*, the findings suggest that the mammals may be exposed to additional species of RF spirochete.

We recommend increased surveillance of small and medium sized mammals within metropolitan areas of Texas. San Antonio, Austin, and Dallas, Texas are in the top 11 most populated cities in the United States, are rapidly expanding, and evidence indicates that *B*. *turicatae* may be emerging in these areas. In 2017 there was an outbreak of TBRF among conference attendees in Austin, Texas [34], which is located in Travis County. This outbreak was in a densely populated area of the city and *B*. *turicatae* infected ticks were collected from rodent dens at a public park near the conference site. In our current report, there was little overlap between the Texas ecoregions that were sampled for the different vertebrate species (Table 1), and only three rodents were collected in Travis County. Future studies should focus on small and medium sized vertebrate sampling in regions where *B*. *turicatae* is emerging, and investigate host competence for the pathogen. As these studies are conducted, a refined understanding of the vertebrate hosts that support the ecology of *B*. *turicatae* will be attained, and surveillance and countermeasures can be implemented to improve public health.
